# Impact of High Serum Lysozyme Activity on Renal Function and Survival Outcomes in Transplant‐Eligible and Ineligible Acute Myeloid Leukemia

**DOI:** 10.1002/cam4.71741

**Published:** 2026-03-23

**Authors:** Takafumi Tsushima, Kosuke Matsuo, Akihiro Shoji, Chiharu Kimeda, Rena Matsumoto, Sonoko Shimoji, Yoshikazu Utsu, Shin‐Ichi Masuda, Nobuyuki Aotsuka

**Affiliations:** ^1^ Department of Hematology and Oncology Japanese Red Cross Narita Hospital Narita Japan

**Keywords:** acute kidney injury, acute myeloid leukemia, high serum lysozyme

## Abstract

Lysozyme‐induced nephropathy is a complication of chronic myelomonocytic leukemia and acute myeloid leukemia (AML). Few studies have discussed the association between blood lysozyme levels at AML onset and AML prognosis, which this study aimed to address. A total of 166 AML patients (72 transplant‐eligible and 94 transplant‐ineligible cases) undergoing chemotherapy at our hospital with recorded serum lysozyme levels were retrospectively included between March 2012 and March 2024. The median age, creatinine, and serum lysozyme at diagnosis were 65 (22–86) years, 0.85 mg/dL (0.43–3.56), and 16.4 μg/mL (0.3–560.0), respectively. Hematopoietic stem cell transplantation was performed in 43.4% of all patients. At initial diagnosis, the lysozyme and creatinine levels were positively correlated (the Spearman rank correlation coefficient: +0.516, *p* < 0.05). Using ROC analysis, the optimal serum lysozyme cutoff values for predicting AKI (KDIGO > 0) at AML onset were determined to be 22.4 μg/mL (AUC, 0.89: 95% CI: 0.847–0.951). The two‐year overall survival (OS) did not differ significantly between patients with lysozyme < 22.4 μg/mL and patients with lysozyme ≥ 22.4 μg/mL (two‐year OS, 39.5% vs. 42.2% [95% CI, 29.2%–49.6% vs. 29.8%–54.1%]; *p* = 0.862). The same trend was observed in the transplantation group. After remission induction, most patients with high‐lysozyme levels showed creatinine level recovery (AML onset: median 1.15 mg/dL, 1 month later: median 0.72 mg/dL, 3 months later: median 0.65 mg/dL). The effect of lysozyme levels on prognosis is limited because renal function recovers in many patients with high‐lysozyme levels after chemotherapy.

## Graphical abstract

At AML initial diagnosis, the lysozyme and creatinine levels were positively correlated. However, the effect of lysozyme levels on AML prognosis is limited, because renal function recovers in many patients with high‐lysozyme levels after chemotherapy.

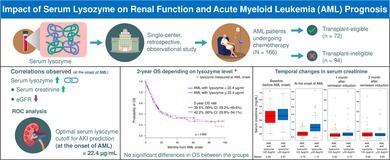

## 1 Introduction

Acute myeloid leukemia (AML) is a hematologic malignancy characterized by the uncontrolled proliferation of neoplastic immature hematopoietic cells resulting from the accumulation of various genetic mutations and chromosomal abnormalities [1]. Acute kidney injury (AKI) may be a prognostic factor for AML [2, 3]. Causes of renal failure in AML include tumor lysis syndrome, acute tubular necrosis, renal infiltration of leukemic cells, sepsis, and chemotherapy‐induced nephrotoxicity. AKI adversely affects the clinical course of patients with AML undergoing induction chemotherapy [3]. Depending on kidney injury severity and the use of dialysis, kidney injury may directly lead to chemotherapy dose reduction.

Lysozymes are small cationic proteins produced by monocytes [4] that are filtered through the glomeruli and reabsorbed in the proximal tubules [5]. In acute myelomonocytic leukemia, acute monocytic leukemia, and chronic myelomonocytic leukemia (CMML), excessive production of lysozyme is observed, which sometimes develops into lysozyme‐induced nephropathy [6, 7, 8]. Lysozyme measurements are often used to diagnose AML, though not all monocytic leukemia cases show elevated lysozyme levels. In 2003, a bimodal relationship between serum lysozyme levels and prognosis was reported in 232 patients with AML [9]. Increased blood lysozyme levels have also been reported in sarcoidosis [10], tuberculosis [11], and inflammatory bowel disease [12].

The basic treatment strategy for AML is intensive combination chemotherapy, palliative chemotherapy, and hematopoietic stem cell transplant aimed at curing the disease. Prognostic factors include white blood cell count at initial diagnosis, chromosomal abnormalities, minimal residual disease (MRD), and patient age [13, 14, 15, 16, 17, 18]. In recent years, there have been almost no reports on renal prognosis and the prognostic value of lysozyme levels for survival in AML, and the cutoff value for serum lysozyme levels in relation to renal injury is unknown. It is also unclear how much benefit chemotherapy has for AML with elevated lysozyme levels. This study retrospectively analyzed the clinical features and prognostic impact of serum lysozyme levels in transplant‐eligible and transplant‐ineligible patients with AML.

## 2 Materials and Methods

### 2.1 Patient Selection

This was a single‐center, retrospective, observational study. A total of 166 patients with AML (72 transplant‐eligible and 94 transplant‐ineligible) undergoing chemotherapy at the Japanese Red Cross Narita Hospital, Narita, Chiba, Japan, were retrospectively included between March 2013 and March 2024.

Patients with acute promyelocytic leukemia, untreated cases, chronic kidney disease (CKD) cases, shock cases at diagnosis, sepsis cases at diagnosis, or those receiving antibiotics at diagnosis were excluded. Antibiotics can sometimes cause kidney damage such as interstitial nephritis. Therefore, we excluded cases that had already received antibiotic treatment prior to referral to our hospital. In our hospital, we reviewed medical checkup results and blood data from patients' primary care physicians at the time of initial leukemia diagnosis for most patients. This allows us to distinguish whether renal failure at the time of leukemia onset is AKI or CKD. A flowchart illustrating patient enrolment is provided in Figure S1.

In our hospital, serum lysozyme levels were only measured at the initial visit (AML onset). This study was conducted according to the principles of the 2013 Declaration of Helsinki and was approved by the ethics committee of the Japanese Red Cross Narita Hospital. As this was a retrospective observational study, written informed consent was not required, and no participants refused to release study documents. Our Institutional Review Board (the ethics committee of the Japanese Red Cross Narita Hospital) approved the opt out method.

### 2.2 Procedures for AKI Evaluation

AKI at diagnosis was defined according to the Kidney Disease: Improving Global Outcomes (KDIGO) clinical practice guideline [19]. Blood lysozyme levels were measured only initially in most cases, and renal function data were collected 1 and 3 months after the start of initial remission induction.

### 2.3 Background Diseases, Treatment Details

AML was diagnosed as ≥ 20% blasts in the bone marrow. Diagnostic criteria were based on the 5th edition of the World Health Organization Classification of Hematolymphoid Tumors: Myeloid and Histiocytic/Dendritic Neoplasms [20]. Intensive chemotherapy was defined as a multiday regimen containing cytarabine at ≥ 100 mg/m^2^/day; regimens that did not meet these criteria, such as those using azacitidine and venetoclax, were defined as palliative chemotherapy [21]. Decisions regarding initial remission induction, consolidation, conditioning, and graft‐versus‐host disease (GVHD) prophylaxis were made at the discretion of the physician.

### 2.4 Statistical Analysis

Progression‐free survival (PFS) was calculated as the time from the date of transplantation or first visit to the date of first progression, relapse, or death without progression. Overall survival (OS) was calculated as the time from the date of transplantation or first visit to the date of death from any cause. Non‐relapse mortality (NRM) was analyzed based on causes of death other than current disease relapse, including infection, veno‐occlusive disease/sinusoidal obstruction syndrome (VOD/SOS), thrombotic microangiopathy (TMA), and GVHD. Baseline clinical characteristics were compared between the groups using the Mann–Whitney *U* test for continuous variables and Fisher's exact test for categorical variables. Survival was analyzed using the Kaplan–Meier method and compared using the log‐rank test to evaluate OS and PFS. Statistical significance was set at a two‐sided *p*‐value < 0.05. The cumulative incidence of relapse and NRM was estimated using Gray's test. The prognostic impact of the variables was evaluated using univariate and multivariate Cox proportional hazards analyses. Using ROC analysis, the optimal serum lysozyme cutoff values for predicting AKI (KDIGO > 0) were determined. All statistical analyses were conducted using EZR (Saitama Medical Center, Jichi Medical University) [22].

## 3 Results

### 3.1 Patient Clinical Characteristics Based on Serum Lysozyme Levels at AML Onset

In our hospital, serum lysozyme levels were only measured at the initial visit (AML onset).

**FIGURE 1:**
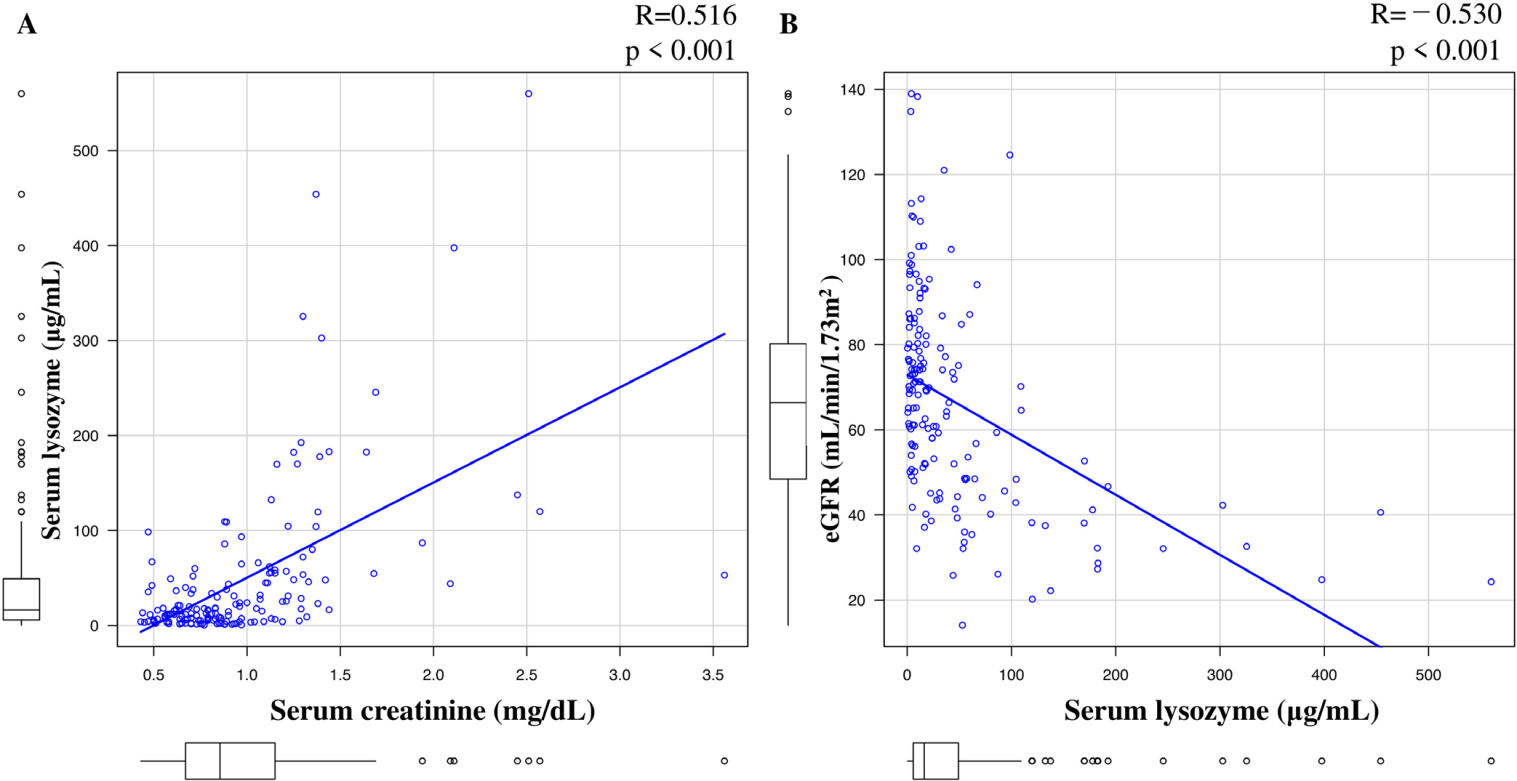
Correlations between serum lysozyme levels and renal parameters. The blue line represents the linear regression showing the correlation between (A) serum creatinine levels and (B) eGFR.

First, we collected lysozyme data and renal function data at the time of leukemia onset. A positive correlation was observed between serum creatinine and lysozyme levels at the initial visit, with a Spearman's rank correlation coefficient of +0.516 (Figure 1A). A negative correlation was observed between estimated glomerular filtration rate and lysozyme, with a Spearman's rank correlation coefficient of −0.53 (Figure 1B). Using ROC analysis, the optimal cutoff value for lysozyme to predict AKI (KDIGO > 0) at AML onset was determined to be 22.4 μg/mL (Figure S2). The clinical characteristics of all patients based on whether the serum lysozyme at AML onset was ≥ or < 22.4 μg/mL are summarized in Table 1.

**TABLE 1:** Clinical characteristics of entire patients according to the first visit serum lysozyme ≥ 22.4 μg/mL or not.

	Total (*N* = 166)	AML with lysozyme < 22.4 μg/mL (*N* = 97)	AML with lysozyme ≧ 22.4 μg/mL (*N* = 69)	*p value*
Age at initial diagnosis, years, median (range)	65 (22–86)	63 (22–86)	67 (24–85)	0.164
Age (initial visit) > 60 years, *N* (%)	99 (59.6)	52 (53.6)	47 (68.1)	0.077
Male sex, *N* (%)	100 (60.2)	55 (56.7)	45 (65.2)	0.335
Therapy‐related AML, *N* (%)	9 (5.4)	5 (5.2)	4 (5.8)	1.000
AML with MRC, *N* (%)	53 (31.9)	36 (37.1)	17 (24.6)	0.095
ELN 2017 risk				0.019
Favorable	20 (12.0)	10 (10.3)	10 (14.5)	
Intermediate	67 (40.4)	32 (33.0)	35 (50.7)	
Adverse	79 (47.6)	55 (56.7)	24 (34.8)	
ELN 2022 risk				0.029
Favorable	24 (14.5)	14 (14.4)	10 (14.5)	
Intermediate	79 (47.6)	40 (41.2)	39 (56.5)	
Adverse	63 (38.0)	45 (46.4)	18 (26.1)	
Laboratory values, median (range)				
WBC/μL, median (range)	18,400 (500–330,700)	12,200 (500–312,800)	64,400 (1500–330,700)	< 0.001
Hb g/dL, median (range)	8.6 (3.1–15.3)	8.4 (3.1–15.3)	8.9 (3.9–13.6)	0.291
Platelet count/μL, median (range)	4.7 (0.3–56.5)	3.9 (0.3–56.5)	5.7 (0.3–30.1)	0.177
peripheral blood blast, median (range)	56.3 (0–99)	47.0 (0–99)	58.0 (0–98)	0.922
Myelomonocytic leukemia/Monocytic leukemia, %	72 (43.4)	15 (15.5)	57 (82.6)	< 0.001
Cre mg/dL, median (range)	0.85 (0.43–3.56)	0.75 (0.43–1.44)	1.15 (0.47–3.56)	< 0.001
eGFR, mL/min/1.73 m^2^, median (range)	66.4 (14.1–139.0)	74.3 (32.1–139.0)	46.7 (14.1–124.6)	< 0.001
KDIGO > 1, %	62 (37.3)	9 (9.3)	53 (76.8)	< 0.001
WT1 expression, copy/μgRNA, median (range)	34,500 (60–700,000)	52,500 (130–700,000)	22,500 (60–340,000)	0.070
Serum lysozyme, μg/mL, median (range)	16.4 (0.3–560.0)	6.9 (0.3–21.2)	55.8 (22.4–560.0)	< 0.001
Induction regimen, *N* (%)				0.606
Intensive chemotherapy	119 (71.7)	3 (20.0)	3 (8.6)	
Palliative chemotherapy	47 (28.3)	2 (13.3)	16 (45.7)	
Allo‐HSCT, *N* (%)	72 (43.4)	46 (47.4)	26 (37.7)	0.266

Abbreviations: Allo‐HSCT, allogeneic hematopoietic stem cell transplantation; AML, acute myeloid leukemia; BUN, blood urea nitrogen; Cre, creatinine; eGFR, estimated glomerular filtration rate; ELN, European Leukemia Network; Hb, hemoglobin; KDIGO, kidney disease improving global outcomes; MRC, myelodysplasia related changes; WBC, white blood cells; WT1, Wilms' tumor gene 1.

The median age of all patients at initial visit was 65.0 years, and 60.2% of them were male. Of all the patients, 72 (43.4%) underwent hematopoietic stem cell transplantation, and 71.7% received intensive chemotherapy as an induction regimen. The minimum serum lysozyme level at the initial visit was 0.3 μg/mL; the maximum value was 560.0 μg/mL, and the median value was 16.4 μg/mL. In the group with high‐lysozyme (≥ 22.4 μg/mL), there was a significant increase in white blood cell count (median: 64,400/μL vs. 12,200/μL) and a significantly higher proportion of monocyte leukemia (82.6% vs. 15.5%).

The clinical characteristics in patients in the transplantation group based on whether or not the serum lysozyme level at the initial visit was ≥ 22.4 μg/mL are summarized in the Table S1. Patients with high‐lysozyme levels tend to have high white blood cell counts and a high incidence of monocyte leukemia. No differences were identified between the two groups in terms of European Leukemia Network (ELN) 2017/2022 risk, disease status at transplantation, conditioning, GVHD prophylaxis, graft source, donor type, GVHD incidence rate, or posttransplant relapse.

### 3.2 Survival Rates of All Patients According to First Visit Serum Lysozyme ≥ or < 22.4 μg/mL, and Changes in Renal Function Over Time After Initial Chemotherapy

**FIGURE 2:**
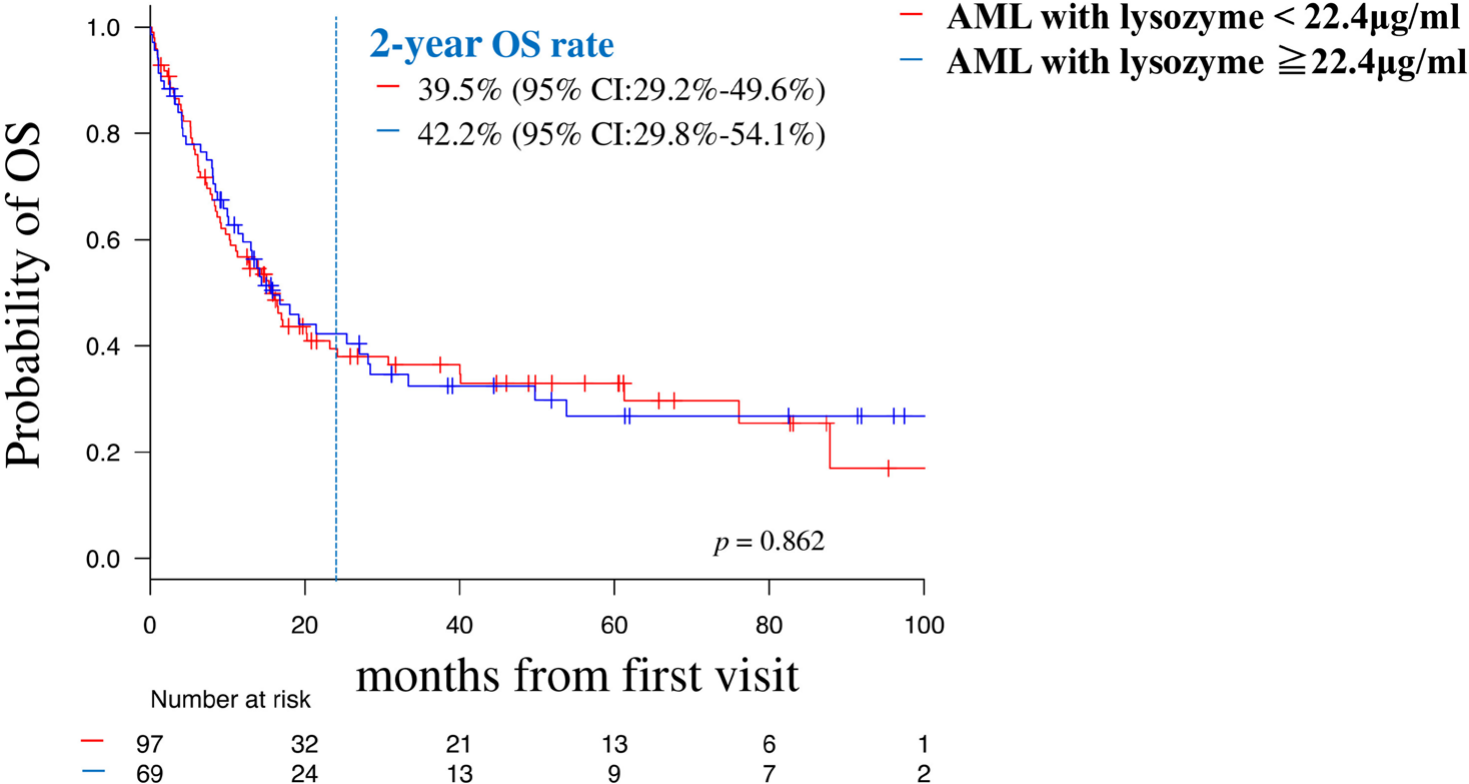
OS of entire patients according to the first visit serum lysozyme or ≥ 22.4 μg/mL. OS, overall survival.

The median follow‐up period was 15.8 (0.1–165.6; standard deviation: 28.56) months, and the two‐year OS rate of all patients was 40.7% (95% confidence interval [CI]: 32.7%–48.4%) (Figure S3). Patients who were adverse risk at first visit had significantly shorter OS and PFS than patients who were not (two‐year OS: 51.9% vs. 28.8%, respectively [95% CI: 40.1–62.5 vs. 18.9–39.4], *p* = 0.004, Figure S4A; 2‐year PFS: 50.0% vs. 26.2% [95% CI: 39.2–59.8 vs. 15.8–37.8], *p* < 0.001, Figure S4B). The two‐year OS did not differ significantly between patients with lysozyme < and ≥ 22.4 μg/mL (two‐year OS: 39.5% vs. 42.2% [95% CI, 29.2%–49.6% vs. 29.8%–54.1%], *p* = 0.862, Figure 2), indicating that the effect of serum lysozyme levels on prognosis was limited.

**FIGURE 3:**
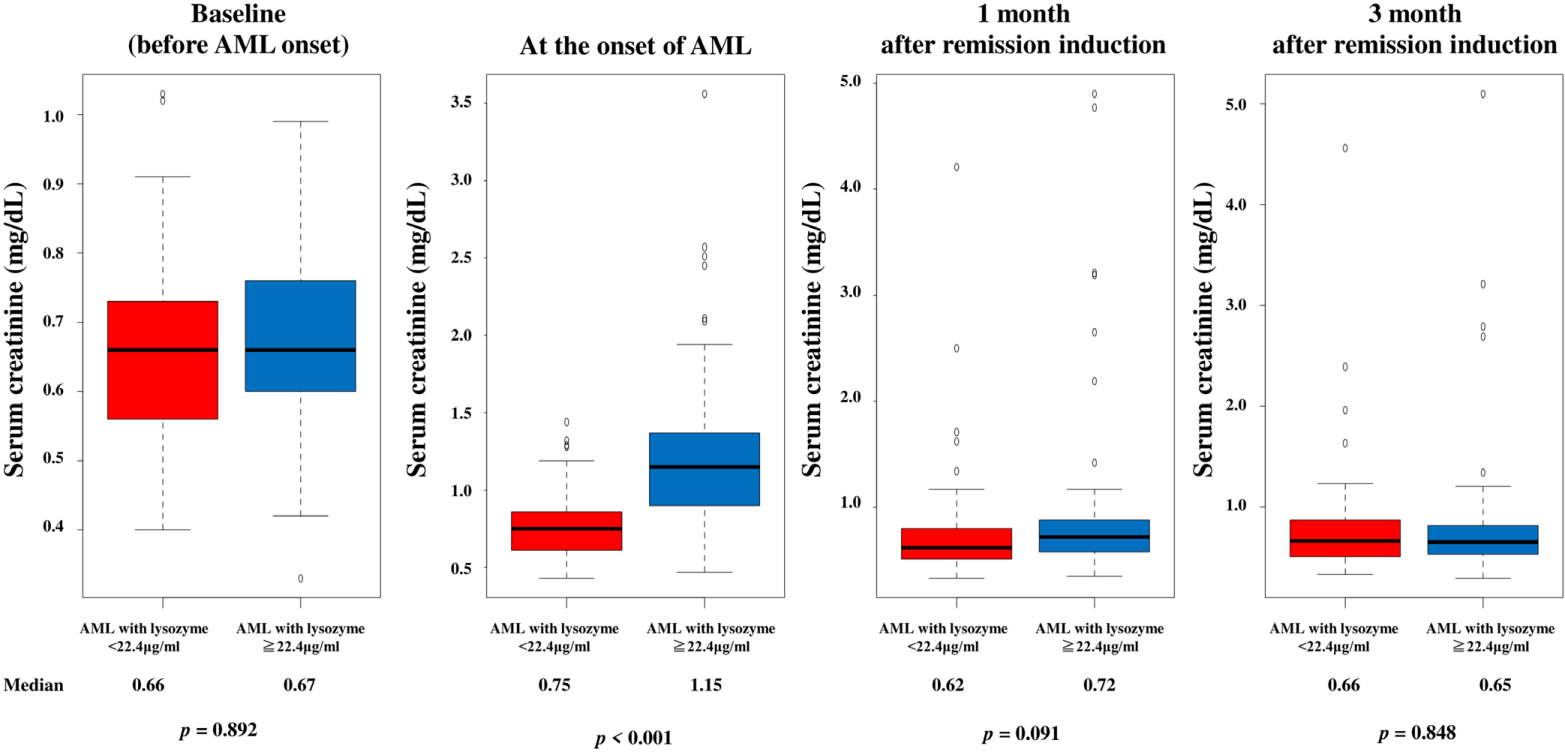
The box plot of the temporal serum creatinine changes in all patients.

Temporal changes in serum creatinine levels are shown as box plots (Figure 3). At the time prior to AML onset, the median serum creatinine level was 0.66 mg/dL in patients with lysozyme < 22.4 μg/mL and 0.67 mg/dL in patients with lysozyme ≥ 22.4 μg/mL (*p* = 0.892). At initial diagnosis, the median serum creatinine level was 0.75 mg/dL in patients with lysozyme < 22.4 μg/mL and 1.15 mg/dL in patients with lysozyme ≥ 22.4 μg/mL (*p* < 0.001). One month after remission induction, the median serum creatinine level was 0.62 mg/dL in patients with lysozyme < 22.4 μg/mL and 0.72 mg/dL in patients with lysozyme ≥ 22.4 μg/mL, with no significant difference in renal function between groups (*p* = 0.091). Three months after remission induction, the median serum creatinine level was 0.66 mg/dL in patients with lysozyme < 22.4 μg/mL and 0.65 mg/dL in patients with lysozyme ≥ 22.4 μg/mL, with no significant difference in renal function (*p* = 0.848).

Temporal changes in creatinine clearance levels are shown as box plots (Figure S5). At the time prior to AML onset, the median creatinine clearance level was 87.2 mL/min in patients with lysozyme < 22.4 μg/mL and 78.1 mL/min in patients with lysozyme ≥ 22.4 μg/mL (*p* = 0.168). At initial diagnosis, the median creatinine clearance level was 82.5 mL/min in patients with lysozyme < 22.4 μg/mL and 50.1 mL/min in patients with lysozyme ≥ 22.4 μg/mL (*p* < 0.001). One month after remission induction, the median creatinine clearance level was 94.6 mL/min in patients with lysozyme < 22.4 μg/mL and 78.8 mL/min in patients with lysozyme ≥ 22.4 μg/mL (*p* = 0.028). Three months after remission induction, the median creatinine clearance level was 85.9 mL/min in patients with lysozyme < 22.4 μg/mL and 88.4 mL/min in patients with lysozyme ≥ 22.4 μg/mL, with no significant difference in renal function (*p* = 0.700).

### 3.3 Survival Rates of Transplant‐Ineligible Patients According to the First Visit Serum Lysozyme < or ≥ 22.4 μg/mL

In transplant‐ineligible patients, the two‐year OS was not significantly different between patients with lysozyme < or ≥ 22.4 μg/mL (two‐year OS: 30.0% vs. 30.0%, respectively [95% CI: 17.2%–43.8% vs. 15.9%–45.4%]; *p* = 0.995; Figure S6A), nor was two‐year PFS (17.3% vs. 21.4% [95% CI: 7.9%–29.6% vs. 9.7%–36.0%]; *p* = 0.498) (Figure S6B), indicating that the effect of serum lysozyme levels on prognosis was limited.

### 3.4 Survival and Relapse Rates of Transplant‐Eligible Patients According to the First Visit Serum Lysozyme<or ≥ 22.4 μg/mL, and Changes in Renal Function Over Time After Initial Chemotherapy

**FIGURE 4:**
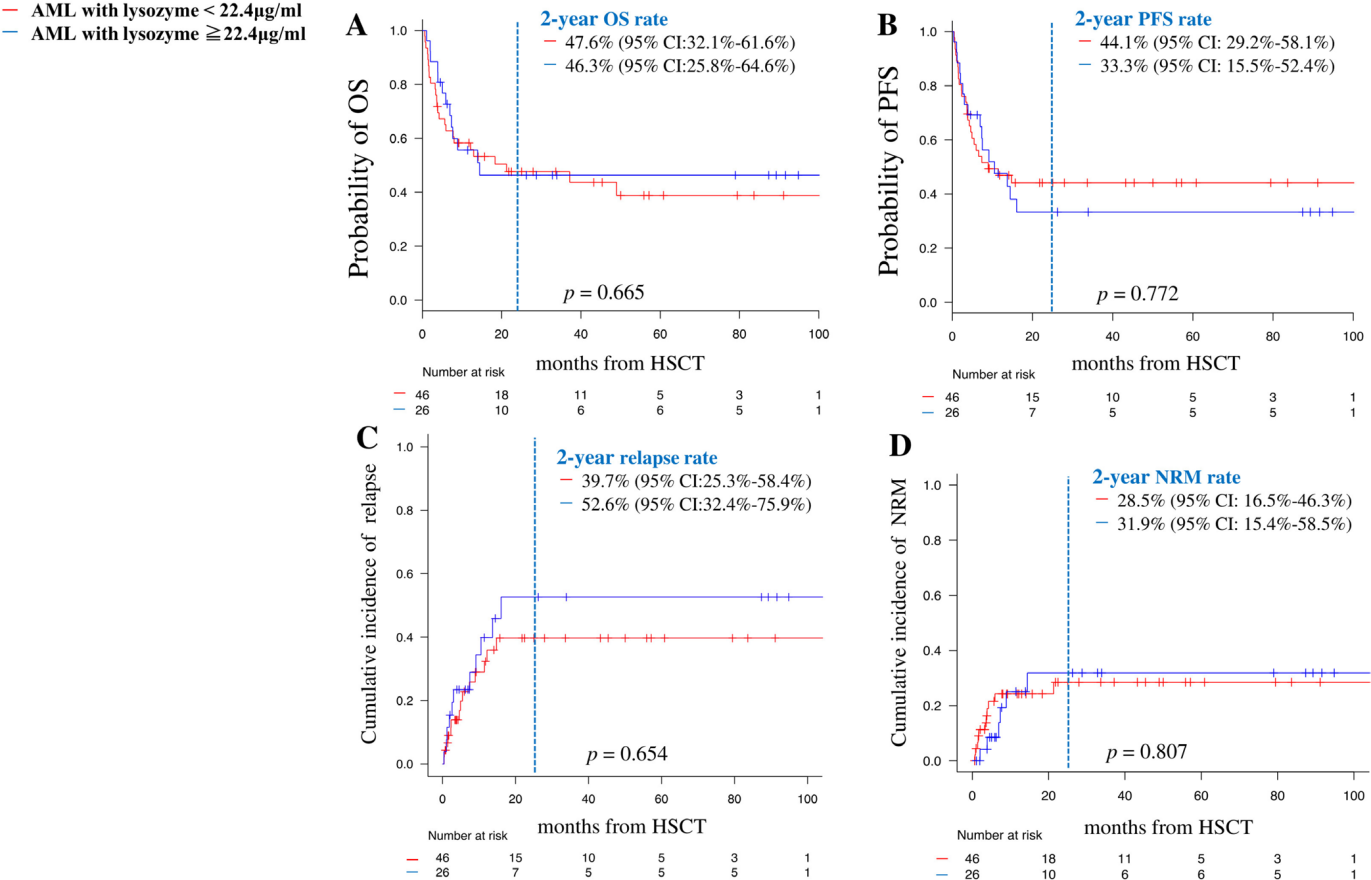
OS (A), PFS (B), cumulative relapse rate (C), and NRM (D) of transplant‐eligible patients according to the first visit serum lysozyme < or ≥ 22.4 μg/mL. OS, overall survival; PFS, progression‐free survival; NRM, non‐relapse mortality.

The two‐year OS rate of transplant‐eligible patients was 47.4% (95% CI: 35.0%–58.8%) (Figure S7A), while the two‐year PFS of transplant‐eligible patients was 40.2% (95% CI: 28.3%–51.7%) (Figure S7B). The cumulative incidence of relapse at 2 years was 45.5% (95% CI: 32.2%–59.2%) (Figure S7C); the cumulative incidence of NRM at 2 years was 29.1% (95% CI: 18.9%–43.2%) (Figure S7D). The two‐year OS was not significantly different between transplant‐eligible patients with lysozyme < or ≥ 22.4 μg/mL (two‐year OS: 47.6% vs. 46.3%, respectively [95% CI: 32.1%–61.6% vs. 25.8%–64.6%]; *p* = 0.665) (Figure 4A), nor was two‐year PFS (44.1% vs. 33.3% [95% CI: 29.2%–58.1% vs. 15.5%–52.4%]; *p* = 0.772) (Figure 4B). The cumulative incidence of relapse was not significantly different between patients with lysozyme < or ≥ 22.4 μg/mL (two‐year relapse rate: 39.7% vs. 52.6% [95% CI: 25.3–58.4 vs. 32.4–75.9]; *p* = 0.654) (Figure 4C). There was no significant difference in the NRM rate between patients with lysozyme < or ≥ 22.4 μg/mL (two‐year cumulative NRM rate: 28.5% vs. 31.9% [95% CI: 16.5–46.3 vs. 15.4–58.5]; *p* = 0.807) (Figure 4D).

Temporal changes in serum creatinine levels are shown as box plots (Figure S8). At initial diagnosis, the median serum creatinine level was 0.66 mg/dL in patients with lysozyme < 22.4 μg/mL and 1.03 mg/dL in patients with lysozyme ≥ 22.4 μg/mL (*p* < 0.001). One month after remission induction, the median serum creatinine level was 0.58 mg/dL in patients with lysozyme < 22.4 μg/mL and 0.58 mg/dL in patients with lysozyme ≥ 22.4 μg/mL, with no significant difference in renal function between groups (*p* = 0.543). Three months after remission induction, the median serum creatinine level was 0.58 mg/dL in patients with lysozyme < 22.4 μg/mL and 0.60 mg/dL in patients with lysozyme ≥ 22.4 μg/mL, with no significant difference in renal function (*p* = 0.971).

### 3.5 Univariate and Multivariate Cox Regression Analyses of OS and PFS

Univariate analysis was performed to identify the factors independently associated with survival in transplant‐ineligible and eligible patients with AML (Table 2). In the transplant‐ineligible group, ELN 2017/2022 adverse genetic risk and being ≥ 60 years old were significantly associated with OS and PFS. In the transplant‐eligible group, acute GVHD Grades II–IV were significantly associated with OS and PFS, and ELN 2022 was significantly associated with PFS.

**TABLE 2:** Univariate and multivariate Cox regression analysis OS and PFS.

Variables	Univariate analysis HR (95% CI)	*p value*	Multivariate analysis HR (95% CI)	*p value*
Not undergoing HSCT OS (from first visit)				
ELN 2017, adverse risk	2.55 (1.54–4.23)	< 0.001		
ELN 2022, adverse risk	2.46 (1.49–4.06)	< 0.001	2.29 (1.37–3.87)	< 0.001
Age (initial visit) > 60 years	3.21 (1.17–8.86)	0.017	2.62 (0.94–7.32)	0.066
Serum lysozyme ≧ 22.4 μg/mL	1.00 (0.62–1.62)	0.995	1.14 (0.69–1.88)	0.587
WBC > 50,000 /μL	0.99 (0.59–1.67)	0.971		
Therapy‐related AML	0.80 (0.24–2.66)	0.715		
Not undergoing HSCT PFS (from first visit)				
ELN 2017 risk, adverse risk	2.52 (1.55–4.11)	< 0.001		
ELN 2022 risk, adverse risk	2.91 (1.77–4.79)	< 0.001	2.61 (1.55–4.39)	< 0.001
Age (initial visit) > 60 years	3.14 (1.26–7.82)	0.009	2.58 (1.02–6.53)	0.045
Serum lysozyme ≧ 22.4 μg/mL	0.85 (0.54–1.36)	0.498	1.00 (0.62–1.63)	0.994
WBC > 50,000 /μL	0.81 (0.48–1.36)	0.418		
Therapy‐related AML	1.12 (0.41–3.08)	0.831		
Undergoing HSCT OS (from HSCT)				
ELN 2017 risk, adverse risk	1.59 (0.81–3.12)	0.169		
ELN 2022 risk, adverse risk	1.80 (0.95–3.43)	0.067	2.00 (1.03–3.85)	0.038
Age (initial visit) > 60 years	1.94 (0.98–3.87)	0.053		
Serum lysozyme ≧ 22.4 μg/mL	0.86 (0.44–1.69)	0.665	0.86 (0.44–1.71)	0.682
Acute GVHD Grade II–IV	1.98 (1.03–3.80)	0.040	2.23 (1.14–4.32)	0.018
Chronic GVHD moderate–severe	1.09 (0.48–2.46)	0.821		
Undergoing HSCT PFS (from HSCT)				
ELN 2017 risk, adverse risk	1.60 (0.85–3.03)	0.142		
ELN 2022 risk, adverse risk	1.91 (1.03–3.54)	0.036	2.25 (1.19–4.27)	0.013
Age (initial visit) > 60 years	1.77 (0.89–3.47)	0.094		
Serum lysozyme ≧ 22.4 μg/mL	1.01 (0.59–2.06)	0.772	1.08 (0.57–2.05)	0.813
Acute GVHD Grade II–IV	1.99 (1.06–3.73)	0.032	2.30 (1.19–4.42)	0.012
Chronic GVHD moderate–severe	1.04 (0.48–2.23)	0.928		

Abbreviations: AML, acute myeloid leukemia; ELN, European Leukemia Network; GVHD, graft‐versus‐host disease; WBC, white blood cells.

In the transplant‐ineligible group, multivariate analysis showed that ELN 2022 adverse risk was significantly associated with OS, and ELN 2022 adverse risk and being ≥ 60 years old were significantly associated with PFS. In the transplant‐eligible group, multivariate analysis showed that ELN 2022 adverse risk and acute GVHD Grades II–IV were significantly associated with OS and PFS.

Even in multivariate analysis, blood lysozyme levels were not significantly associated with prognosis.

## 4 Discussion

The treatment of newly diagnosed AML and renal failure presents several challenges. Cooperation between hematologists and nephrologists is needed, and the management of TLS is important, especially during the initial induction of remission [23]. Some reports suggest that a pretreatment serum creatinine level > 1.4 mg/dL greatly increases the risk of TLS [24]. When leukocyte counts are too high, leukapheresis may be considered [25]. Our analysis indicates that elevated lysozyme levels are associated with renal impairment. Many patients with AKI with high‐lysozyme levels recovered after chemotherapy, suggesting that it is important to start chemotherapy for AML as soon as possible when renal failure and high‐lysozyme levels are observed at initial diagnosis.

If lysozyme‐induced nephropathy is suspected, the presence of lysozyme can be estimated by comparing urinary albumin and total protein concentrations because of the presence of excess lysozyme in urine [26]; this is because lysozymuria is typically non‐albuminuric and appears as a cationic peak on urinary protein electrophoresis [4, 27]. Urinary protein electrophoresis can also be helpful in the treatment of lysozyme nephropathy. Occasionally, nephropathy associated with monocytic leukemia may coexist with monocytic tubulointerstitial infiltration or lysozyme nephropathy, complicating the pathogenesis [26]. Unfortunately, despite CMML data, the prognosis for patients with glomerular involvement is poor, with gradual worsening of renal function despite treatment that often eventually leads to renal failure [28]; this suggests the coexistence of glomerular lesions in cases where renal function is poorly improved with conventional chemotherapy.

High serum lysozyme levels have been reported to be a poor prognostic factor in CMML [29]. In CMML, serum lysozyme cutoff values related to AKI have not been established. Early cytoreduction, renal risk factor management, and volume resuscitation can potentially improve renal function in lysozyme‐induced nephropathy [30, 31]. We estimated the lysozyme levels that induce AKI (KDIGO > 0) and found that serum lysozyme levels of 22.4 μg/mL or higher may cause AKI. However, high‐lysozyme levels were not a prognostic factor in our cohort. This may be because many patients with AML with high‐lysozyme levels recovered quickly from AKI after chemotherapy was initiated. However, an increase in lysozyme levels does not necessarily equate to an increase in tumor volume, as it may also indicate an immune response to the tumor as well as overproduction by the tumor [32].

Few reports have shown how lysozymes are related to the prognosis of transplant‐eligible AML. In our study, compared to the non‐elevated lysozyme group, the elevated lysozyme group also had a similar CR before transplantation and a similar survival rate, indicating no adverse effects of elevated lysozyme in transplant‐eligible AML. We also examined the possibility that patients with high‐lysozyme levels have concomitant renal impairment, which may lead to elevated TMA and VOD/SOS during transplantation; there was no predominant increase in NRM related to VOD/SOS or TMA in the high‐lysozyme group of transplant‐eligible AML, and this high‐level lysozyme was not a negative factor in transplant‐eligible AML.

The limitations of this study include the fact that direct tumor invasion and lysozyme nephropathy were not evaluated in all patients through renal biopsy; additionally, there were no quantitative data on proteinuria or posttreatment lysozyme data. In our hospital, serum lysozyme levels were only measured at the initial visit (AML onset). Since lysozyme measurement is used for diagnosing leukemia subtypes, we do not have follow‐up data. Urinary lysozyme may more specifically indicate acute kidney injury caused by lysozyme; however, we do not have data. Renal biopsies were also not performed in our cohort. Although renal biopsy remains the gold standard for the diagnosis of AKI in AML, clinicians should be aware of the clinical course of lysozyme‐induced nephropathy from lysozyme elevation and AKI, as renal biopsy sometimes cannot be safely performed because of thrombocytopenia and dyscoagulation associated with AML. Another limitation is the heterogeneity of the treatment owing to the long study period. An additional limitation of this cohort was the exclusion of patients with CKD from the analysis, as AML can also occur in this group.

In conclusion, serum lysozyme levels were correlated with renal function at initial diagnosis. However, the impact of lysozyme levels on the prognosis of transplant‐eligible and transplant‐ineligible patients with AML is limited. To confirm our findings, an independent prospective study with a larger sample size and more detailed investigation is required.

## Author Contributions

**Takafumi Tsushima:** conceptualization, validation, formal analysis, project administration, visualization, writing – original draft, writing – review and editing, investigation, methodology, software, data curation. **Kosuke Matsuo:** supervision. **Akihiro Shoji:** supervision. **Chiharu Kimeda:** supervision. **Rena Matsumoto:** supervision. **Sonoko Shimoji:** supervision. **Yoshikazu Utsu:** supervision. **Shin‐Ichi Masuda:** supervision. **Nobuyuki Aotsuka:** supervision.

## Funding

The authors have nothing to report.

## Ethics Statement

All procedures were performed in accordance with the ethical standards of the institutional and national research committees and the 1964 Helsinki declaration and its later amendments or comparable ethical standards. This study was approved by the Ethics Committee of the Japanese Red Cross Narita Hospital.

## Consent

The authors have nothing to report.

## Conflicts of Interest

The authors declare no conflicts of interest.

## Supporting information


**Figure S1:** Study flow chart illustrating the patient enrollment process.
**Figure S2:** Receiver operating curve analysis to determine the optimal cutoff for lysozyme to predict AKI (KDIGO > 0).
**Figure S3:** OS (A) of entire patients. OS, overall survival.
**Figure S4:** OS of entire patients according to the positivity of 2017 ELN/2022 ELN adverse risk at first visit. OS, overall survival; ELN, European Leukemia Network.
**Figure S5:** The box plot of the temporal creatinine clearance changes in all patients.
**Figure S6:** OS (A) and PFS (B) of transplant‐ineligible patients according to the first visit serum lysozyme < or ≥ 22.4 μg/mL. OS, overall survival; PFS, progression‐free survival.
**Figure S7:** OS (A), PFS (B), cumulative relapse rate (C), and NRM (D) of transplant‐eligible patients. OS, overall survival; PFS, progression‐free survival; NRM, non‐relapse mortality.
**Figure S8:** The box plot of the temporal serum creatinine changes in transplant‐eligible patients.
**Table S1:** Clinical characteristics of transplant‐eligible patients according to the first visit serum lysozyme ≥ 22.4 μg/mL or not.

## Data Availability

The data that support the findings of this study are available from the corresponding author upon reasonable request.
