# Supplementary material for: Impact of High Serum Lysozyme Activity on Renal Function and Survival Outcomes in Transplant‐Eligible and Ineligible Acute Myeloid Leukemia
Source: Cancer Med. 2026 Mar 23;15(3):e71741. doi: 10.1002/cam4.71741 (PMC13140699; doi:10.1002/cam4.71741)
Supplement: Supplementary file 1 — Figure S1: Study flow chart illustrating the patient enrollment process. Figure S2: Receiver operating curve analysis to determine the optimal cutoff for lysozyme to predict AKI (KDIGO > 0). Figure S3: OS (A) of entire patients. OS, overall survival. Figure S4: OS of entire patients according to the positivity of 2017 ELN/2022 ELN adverse risk at first visit. OS, overall survival; ELN, European Leukemia Network. Figure S5: The box plot of the temporal creatinine clearance changes in all patients. Figure S6: OS (A) and PFS (B) of transplant‐ineligible patients according to the first visit serum lysozyme < or ≥ 22.4 μg/mL. OS, overall survival; PFS, progression‐free survival. Figure S7: OS (A), PFS (B), cumulative relapse rate (C), and NRM (D) of transplant‐eligible patients. OS, overall survival; PFS, progression‐free survival; NRM, non‐relapse mortality. Figure S8: The box plot of the temporal serum creatinine changes in transplant‐eligible patients. Table S1: Clinical characteristics of transplant‐eligible patients according to the first visit serum lysozyme ≥ 22.4 μg/mL or not. [file CAM4-15-e71741-s001.zip › cam471741-sup-0009-TableS1.docx]

**Supplementary Table 1.**

Clinical characteristics of transplant-eligible patients according to the first visit serum lysozyme ≧22.4μg/mL or not

|  | **Total** | **AML with lysozyme**  **< 22.4μg/ml** | **AML with lysozyme**  ≧**22.4μg/ml** | ***p*.value** |
| --- | --- | --- | --- | --- |
|  | (N=72) | (N=46) | (N=26) |  |
| **Age at initial diagnosis, years, median (range)** | 52 (22-73) | 51.5 (22-73) | 52.5 (24-69) | 0.598 |
| **Age (initial visit) > 60 years, N (%)** | 17 (23.6) | 9 (19.6) | 8 (30.8) | 0.387 |
| **Male sex, N (%)** | 38 (52.8) | 20 (43.5) | 18 (69.2) | 0.051 |
| **Therapy-related AML, N (%)** | 5 (6.9) | 3 (6.5) | 2 (7.7) | 1.000 |
| **AML with MRC, N (%)** | 18 (25.0) | 14 (30.4) | 4 (15.4) | 0.257 |
| **ELN 2017 risk** | |  |  | 0.081 |
| Favorable | 9 (12.5) | 5 (10.9) | 4 (15.4) |  |
| Intermediate | 23 (31.9) | 11 (23.9) | 12 (46.2) |  |
| Adverse | 40 (55.6) | 30 (65.2) | 10 (38.5) |  |
| **ELN 2022 risk** | |  |  | 0.547 |
| Favorable | 9 (12.5) | 5 (10.9) | 4 (15.4) |  |
| Intermediate | 32 (44.4) | 19 (41.3) | 13 (50.0) |  |
| Adverse | 31 (43.1) | 22 (47.8) | 9 (34.6) |  |
| **Laboratory values (initial visit), median (range)** | |  |  |  |
| WBC /μL, median (range) | 28300 (500-312800) | 14000 (500-312800) | 78750 (1500-239500) | <0.001 |
| Hb g/dL, median (range) | 8.6 (4.5-13.3) | 8.45 (4.8-13.2) | 9.30 (4.5-13.3) | 0.622 |
| Platelet count /μL, median (range) | 3.2 (0.6-38.9) | 3.8 (0.9-38.9) | 3.1 (0.6-30.1) | 0.967 |
| peripheral blood blast, median (range) | 63 (2-99) | 67.9 (2-99) | 62.5 (10-98) | 0.828 |
| Myelomonocytic leukaemia / Monocytic leukaemia, % | 26 (36.1) | 5 (10.9) | 21 (80.8) | <0.001 |
| Cre mg/dL, median (range) | 0.74  (0.43-3.56) | 0.66  (0.43-0.97) | 1.04  (0.47-3.56) | <0.001 |
| eGFR, mL/min/1.73m^2^, median (range) | 75.4  (14.1-139.0) | 81.9  (56.7-139.0) | 60.1  (14.1-124.6) | <0.001 |
| KDIGO>1, % | 17 (23.6) | 0 (0.0) | 17 (65.4) | <0.001 |
| WT1 expression, copy/μgRNA, median (range) | 79500  (60-700000) | 95000  (210-700000) | 34000  (60-340000) | 0.163 |
| Serum lysozyme, μg/mL, median (range) | 12.5  (0.3-560.0) | 4.9  (0.3-21.0) | 52.5  (24.0-560.0) | <0.001 |
| **Induction regimen, N (%)** | |  |  | 0.702 |
| Intensive chemotherapy | 64 (88.9) | 40 (87.0) | 24 (92.3) |  |
| Palliative chemotherapy | 8 (11.1) | 6 (13.0) | 2 (7.7) |  |
| **Disease status at transplantation, N (%)** | | |  | 1.000 |
| hematological CR | 42 (58.3) | 27 (58.7) | 15 (57.7) |  |
| not hematological CR | 30 (41.6) | 19 (41.3) | 11 (42.3) |  |
| **Conditioning, N (%)** | |  |  | 0.526 |
| MAC | 59 (81.9) | 39 (84.8) | 20 (76.9) |  |
| RIC | 13 (18.0) | 7 (15.2) | 6 (23.1) |  |
| **Prophylaxis of GVHD, N (%)** | |  |  | 0.797 |
| ATG+mPSL+CNI | 17 (23.6) | 12 (26.0) | 5 (19.2) |  |
| PTCY + TAC + MMF | 7 (9.7) | 5 (10.9) | 2 (7.7) |  |
| short MTX + CNI | 35 (48.6) | 19 (41.3) | 16 (61.5) |  |
| MMF + CNI | 13 (18.1) | 10 (21.7) | 3 (11.5) |  |
| **Graft source, N (%)** | |  |  | 0.762 |
| PB | 36 (50.0) | 24 (52.2) | 11 (42.3) |  |
| BM | 13 (18.0) | 8 (17.4) | 5 (19.2) |  |
| CB | 23 (31.9) | 13 (28.2) | 10 (38.5) |  |
| **Donor type, N** |  |  |  | 0.863 |
| Haploidentical | 24 (33.3) | 17 (37.0) | 7 (26.9) |  |
| Matched related donor (MRD) | 9 (12.5) | 6 (13.0) | 3 (11.5) |  |
| Matched unrelated donor (MUD) | 10 (13.8) | 6 (13.0) | 4 (15.4) |  |
| Mismatched related donor (MMRD) | 1 (1.3) | 1 (2.2) | 0 (0.0) |  |
| Mismatched unrelated donor (MMUD) | 28 (38.8) | 16 (34.8) | 12 (46.2) |  |
| **Acute GVHD gradeⅡ-Ⅳ, N (%)** | 33 (45.8) | 20 (43.5) | 13 (50.0) | 0.630 |
| **Chronic GVHD moderate-severe, N (%)** | 21 (29.1) | 13 (28.3) | 8 (30.8) | 0.752 |
| **TMA or VOD/SOS, N (%)** | 8 (11.1) | 6 (13.0) | 2 (7.7) | 0.702 |
| **Post-transplant relapse, N (%)** | 25 (34.7) | 14 (30.4) | 11 (42.3) | 0.318 |

Abbreviations; AML, acute myeloid leukaemia; ATG, antithymocyte globulin; BM, bone marrow; BUN, blood urea nitrogen; CB, cord blood; CNI, calcineurin inhibitor; CR, complete remission; Cre, creatinine; ELN, european leukaemia network; eGFR, estimated glomerular filtration rate; GVHD, graft vs host disease; Hb, hemoglobin; KDIGO, kidney disease improving global outcomes; MAC, myeloablative conditioning; mPSL, methylprednisolone; MRC, myelodysplasia related changes; MTX, methotrexate; PB, peripheral blood; PTCY, post cyclosphosphamide; RIC, reduced intensity conditioning; SOS, sinusoidal obstruction syndrome; TAC, tacrolimus; TMA, thrombotic microangiopathy; VOD, veno-occlusive disease; WBC, white blood cells; WT1, Wilms tumor gene 1.
