# Supplementary figures and images for: Impact of High Serum Lysozyme Activity on Renal Function and Survival Outcomes in Transplant‐Eligible and Ineligible Acute Myeloid Leukemia
Source: Cancer Med. 2026 Mar 23;15(3):e71741. doi: 10.1002/cam4.71741 (PMC13140699; doi:10.1002/cam4.71741)

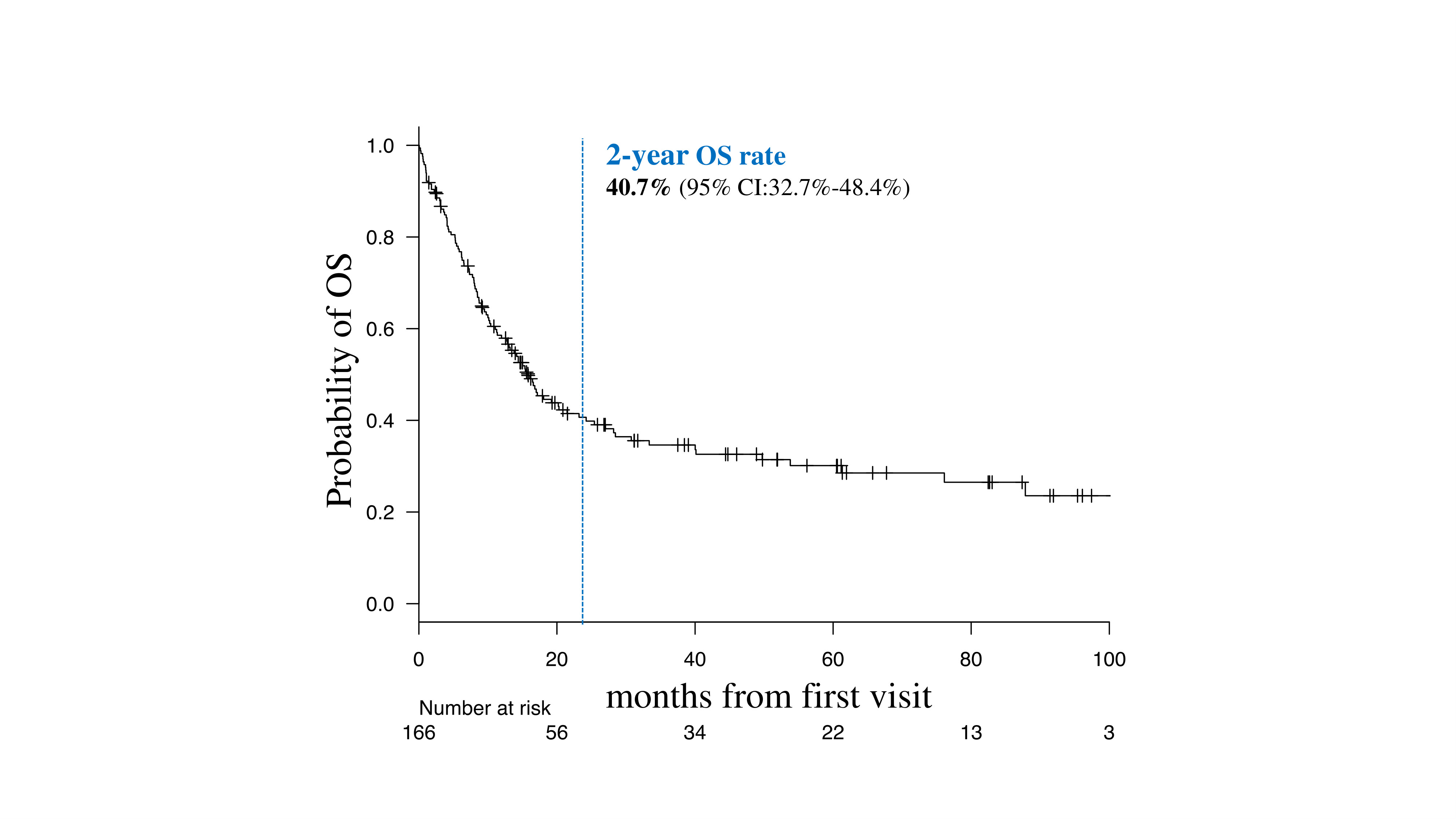

Supplement: Supplementary file 1 — Figure S1: Study flow chart illustrating the patient enrollment process. Figure S2: Receiver operating curve analysis to determine the optimal cutoff for lysozyme to predict AKI (KDIGO > 0). Figure S3: OS (A) of entire patients. OS, overall survival. Figure S4: OS of entire patients according to the positivity of 2017 ELN/2022 ELN adverse risk at first visit. OS, overall survival; ELN, European Leukemia Network. Figure S5: The box plot of the temporal creatinine clearance changes in all patients. Figure S6: OS (A) and PFS (B) of transplant‐ineligible patients according to the first visit serum lysozyme < or ≥ 22.4 μg/mL. OS, overall survival; PFS, progression‐free survival. Figure S7: OS (A), PFS (B), cumulative relapse rate (C), and NRM (D) of transplant‐eligible patients. OS, overall survival; PFS, progression‐free survival; NRM, non‐relapse mortality. Figure S8: The box plot of the temporal serum creatinine changes in transplant‐eligible patients. Table S1: Clinical characteristics of transplant‐eligible patients according to the first visit serum lysozyme ≥ 22.4 μg/mL or not. [file CAM4-15-e71741-s001.zip › cam471741-sup-0003-FigureS3.jpg]

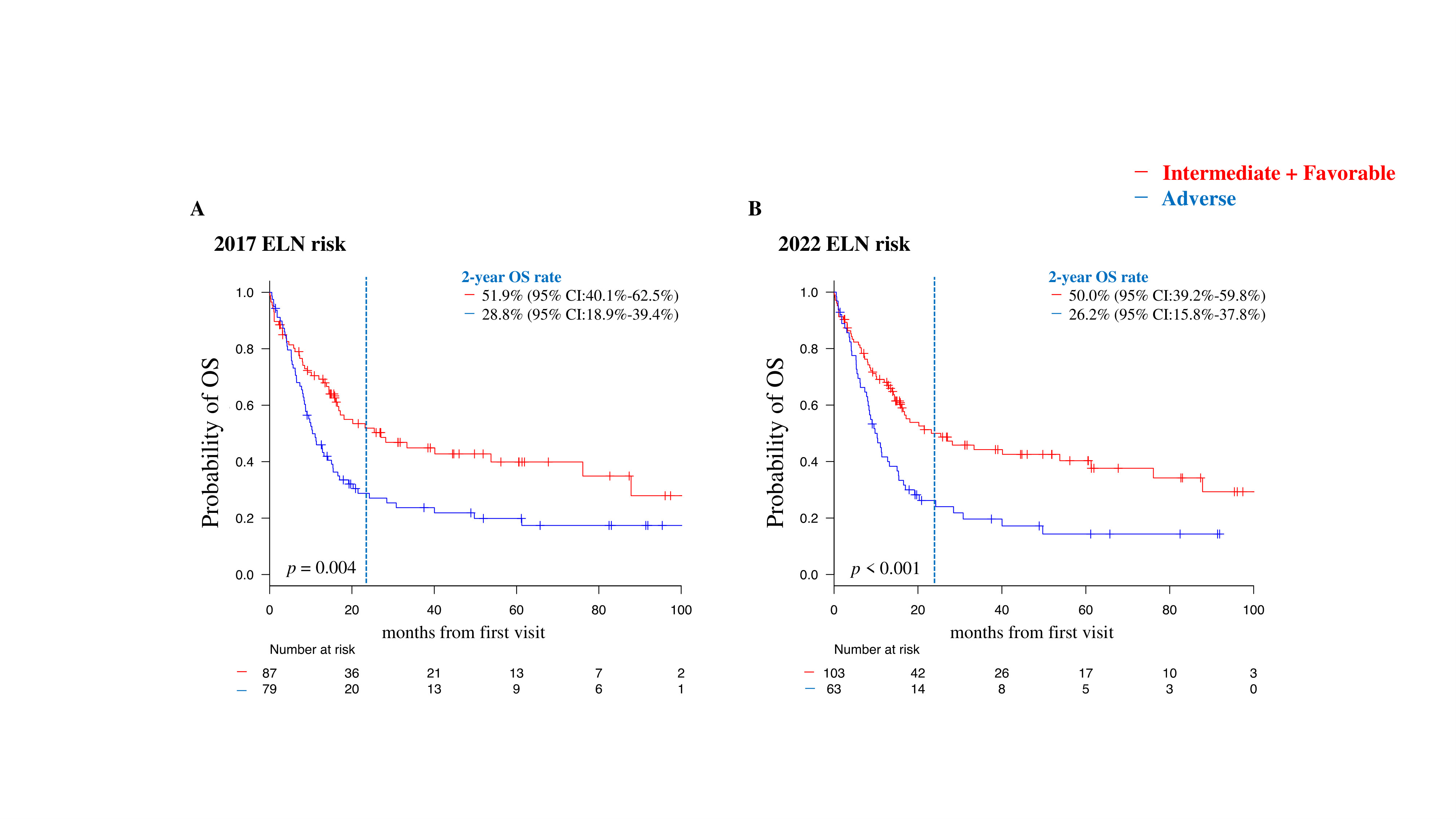

Supplement: Supplementary file 1 — Figure S1: Study flow chart illustrating the patient enrollment process. Figure S2: Receiver operating curve analysis to determine the optimal cutoff for lysozyme to predict AKI (KDIGO > 0). Figure S3: OS (A) of entire patients. OS, overall survival. Figure S4: OS of entire patients according to the positivity of 2017 ELN/2022 ELN adverse risk at first visit. OS, overall survival; ELN, European Leukemia Network. Figure S5: The box plot of the temporal creatinine clearance changes in all patients. Figure S6: OS (A) and PFS (B) of transplant‐ineligible patients according to the first visit serum lysozyme < or ≥ 22.4 μg/mL. OS, overall survival; PFS, progression‐free survival. Figure S7: OS (A), PFS (B), cumulative relapse rate (C), and NRM (D) of transplant‐eligible patients. OS, overall survival; PFS, progression‐free survival; NRM, non‐relapse mortality. Figure S8: The box plot of the temporal serum creatinine changes in transplant‐eligible patients. Table S1: Clinical characteristics of transplant‐eligible patients according to the first visit serum lysozyme ≥ 22.4 μg/mL or not. [file CAM4-15-e71741-s001.zip › cam471741-sup-0004-FigureS4.jpg]

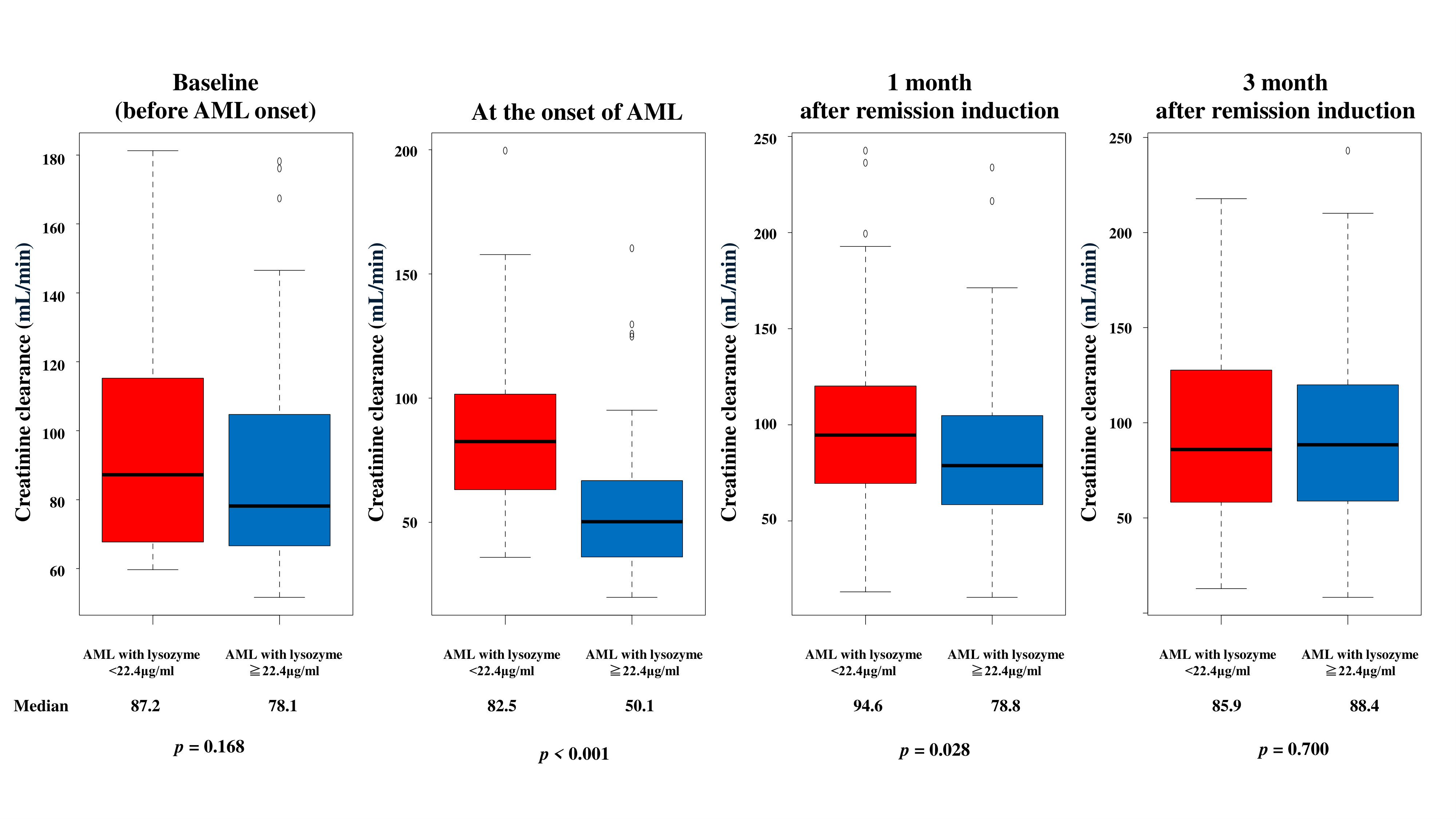

Supplement: Supplementary file 1 — Figure S1: Study flow chart illustrating the patient enrollment process. Figure S2: Receiver operating curve analysis to determine the optimal cutoff for lysozyme to predict AKI (KDIGO > 0). Figure S3: OS (A) of entire patients. OS, overall survival. Figure S4: OS of entire patients according to the positivity of 2017 ELN/2022 ELN adverse risk at first visit. OS, overall survival; ELN, European Leukemia Network. Figure S5: The box plot of the temporal creatinine clearance changes in all patients. Figure S6: OS (A) and PFS (B) of transplant‐ineligible patients according to the first visit serum lysozyme < or ≥ 22.4 μg/mL. OS, overall survival; PFS, progression‐free survival. Figure S7: OS (A), PFS (B), cumulative relapse rate (C), and NRM (D) of transplant‐eligible patients. OS, overall survival; PFS, progression‐free survival; NRM, non‐relapse mortality. Figure S8: The box plot of the temporal serum creatinine changes in transplant‐eligible patients. Table S1: Clinical characteristics of transplant‐eligible patients according to the first visit serum lysozyme ≥ 22.4 μg/mL or not. [file CAM4-15-e71741-s001.zip › cam471741-sup-0005-FigureS5.jpg]

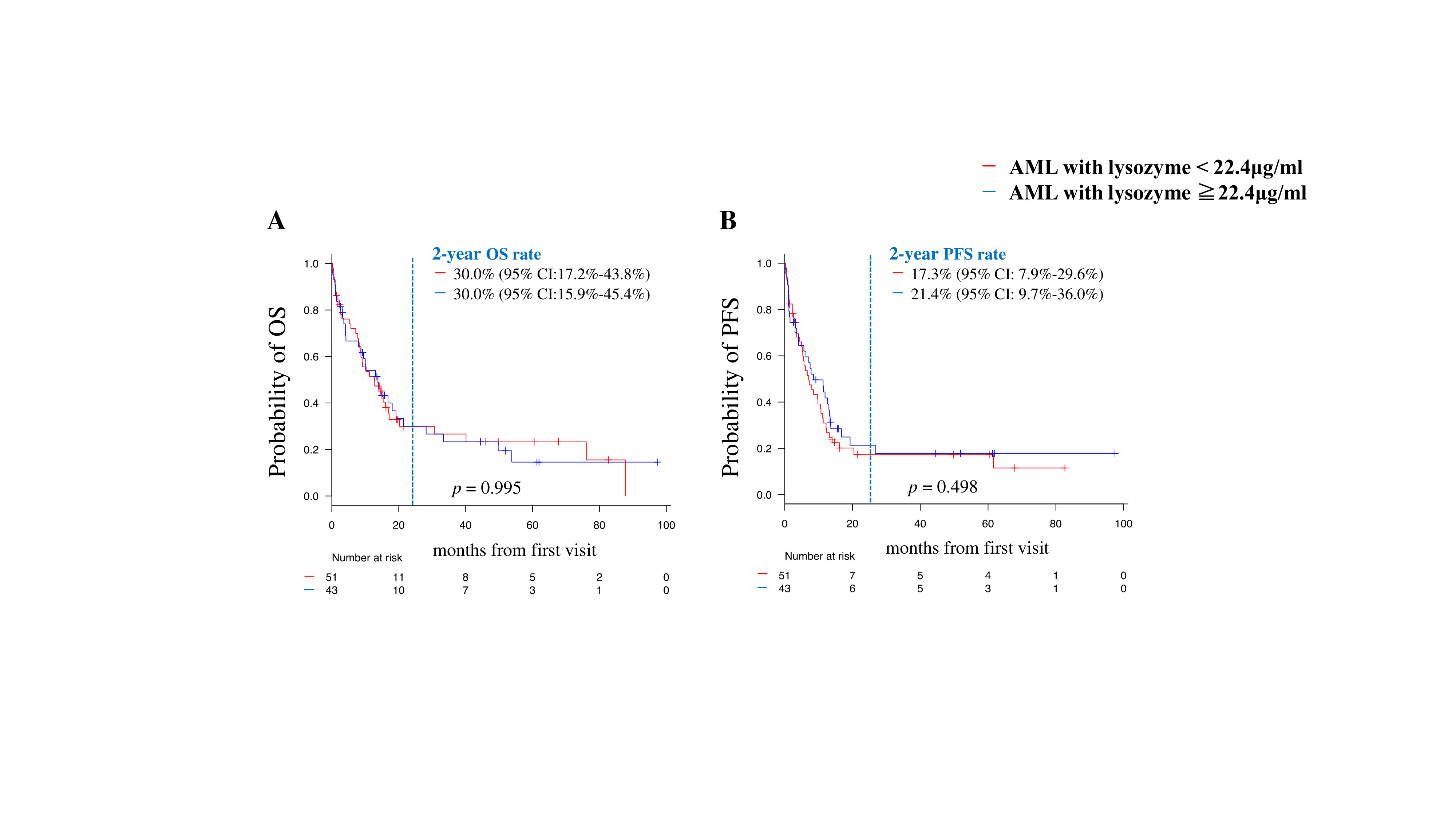

Supplement: Supplementary file 1 — Figure S1: Study flow chart illustrating the patient enrollment process. Figure S2: Receiver operating curve analysis to determine the optimal cutoff for lysozyme to predict AKI (KDIGO > 0). Figure S3: OS (A) of entire patients. OS, overall survival. Figure S4: OS of entire patients according to the positivity of 2017 ELN/2022 ELN adverse risk at first visit. OS, overall survival; ELN, European Leukemia Network. Figure S5: The box plot of the temporal creatinine clearance changes in all patients. Figure S6: OS (A) and PFS (B) of transplant‐ineligible patients according to the first visit serum lysozyme < or ≥ 22.4 μg/mL. OS, overall survival; PFS, progression‐free survival. Figure S7: OS (A), PFS (B), cumulative relapse rate (C), and NRM (D) of transplant‐eligible patients. OS, overall survival; PFS, progression‐free survival; NRM, non‐relapse mortality. Figure S8: The box plot of the temporal serum creatinine changes in transplant‐eligible patients. Table S1: Clinical characteristics of transplant‐eligible patients according to the first visit serum lysozyme ≥ 22.4 μg/mL or not. [file CAM4-15-e71741-s001.zip › cam471741-sup-0006-FigureS6.jpg]

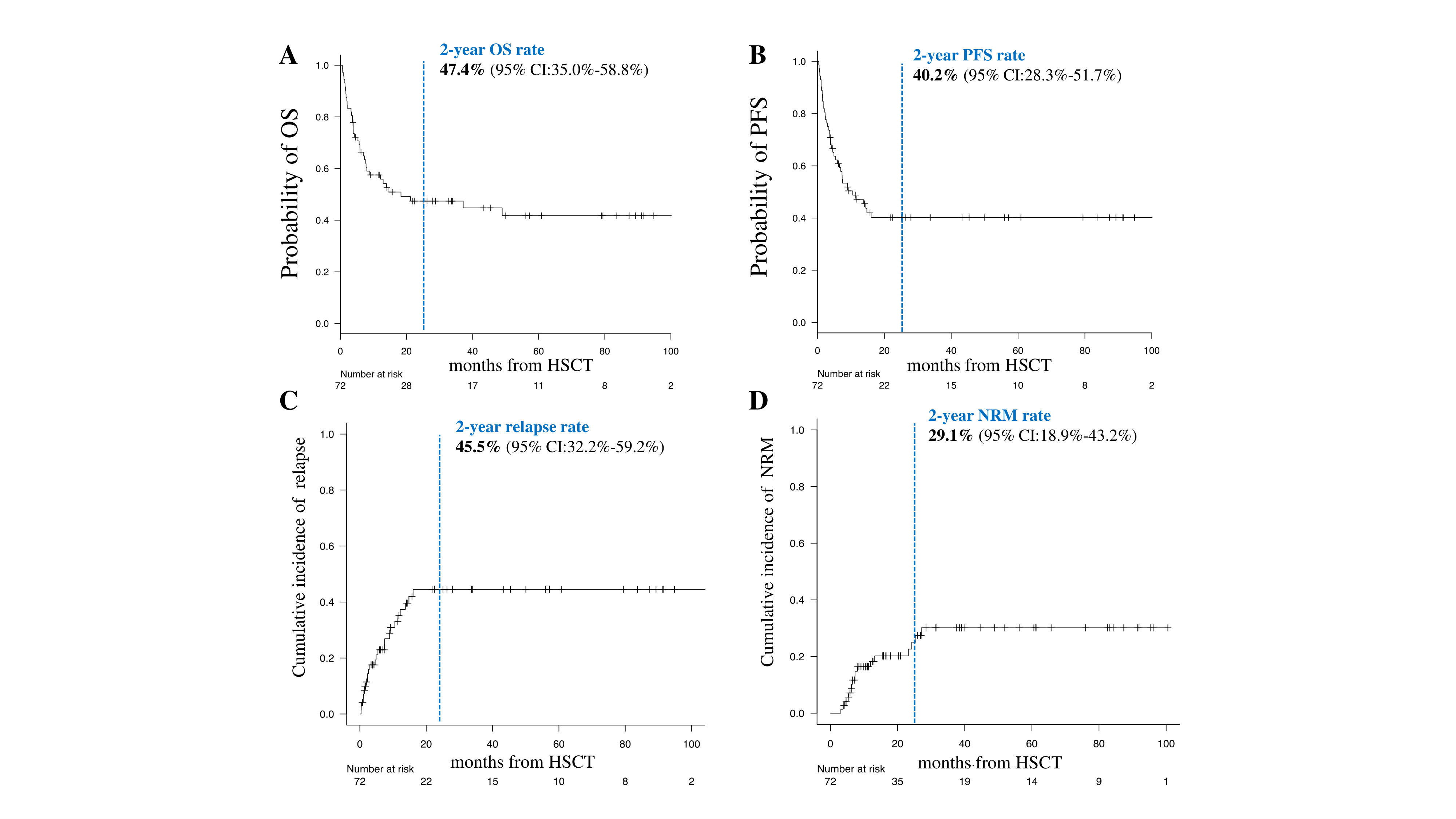

Supplement: Supplementary file 1 — Figure S1: Study flow chart illustrating the patient enrollment process. Figure S2: Receiver operating curve analysis to determine the optimal cutoff for lysozyme to predict AKI (KDIGO > 0). Figure S3: OS (A) of entire patients. OS, overall survival. Figure S4: OS of entire patients according to the positivity of 2017 ELN/2022 ELN adverse risk at first visit. OS, overall survival; ELN, European Leukemia Network. Figure S5: The box plot of the temporal creatinine clearance changes in all patients. Figure S6: OS (A) and PFS (B) of transplant‐ineligible patients according to the first visit serum lysozyme < or ≥ 22.4 μg/mL. OS, overall survival; PFS, progression‐free survival. Figure S7: OS (A), PFS (B), cumulative relapse rate (C), and NRM (D) of transplant‐eligible patients. OS, overall survival; PFS, progression‐free survival; NRM, non‐relapse mortality. Figure S8: The box plot of the temporal serum creatinine changes in transplant‐eligible patients. Table S1: Clinical characteristics of transplant‐eligible patients according to the first visit serum lysozyme ≥ 22.4 μg/mL or not. [file CAM4-15-e71741-s001.zip › cam471741-sup-0007-FigureS7.jpg]

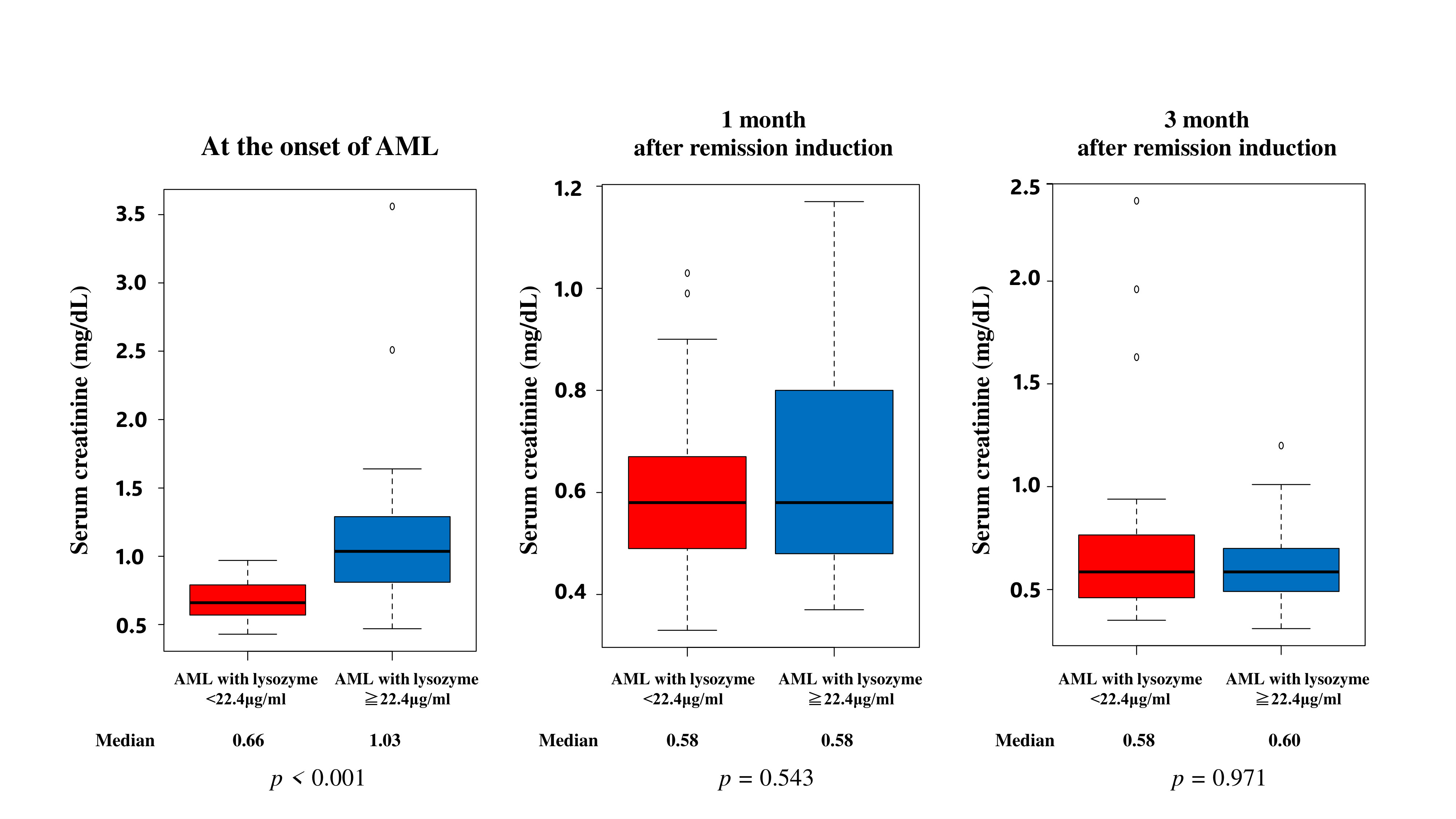

Supplement: Supplementary file 1 — Figure S1: Study flow chart illustrating the patient enrollment process. Figure S2: Receiver operating curve analysis to determine the optimal cutoff for lysozyme to predict AKI (KDIGO > 0). Figure S3: OS (A) of entire patients. OS, overall survival. Figure S4: OS of entire patients according to the positivity of 2017 ELN/2022 ELN adverse risk at first visit. OS, overall survival; ELN, European Leukemia Network. Figure S5: The box plot of the temporal creatinine clearance changes in all patients. Figure S6: OS (A) and PFS (B) of transplant‐ineligible patients according to the first visit serum lysozyme < or ≥ 22.4 μg/mL. OS, overall survival; PFS, progression‐free survival. Figure S7: OS (A), PFS (B), cumulative relapse rate (C), and NRM (D) of transplant‐eligible patients. OS, overall survival; PFS, progression‐free survival; NRM, non‐relapse mortality. Figure S8: The box plot of the temporal serum creatinine changes in transplant‐eligible patients. Table S1: Clinical characteristics of transplant‐eligible patients according to the first visit serum lysozyme ≥ 22.4 μg/mL or not. [file CAM4-15-e71741-s001.zip › cam471741-sup-0008-FigureS8.jpg]

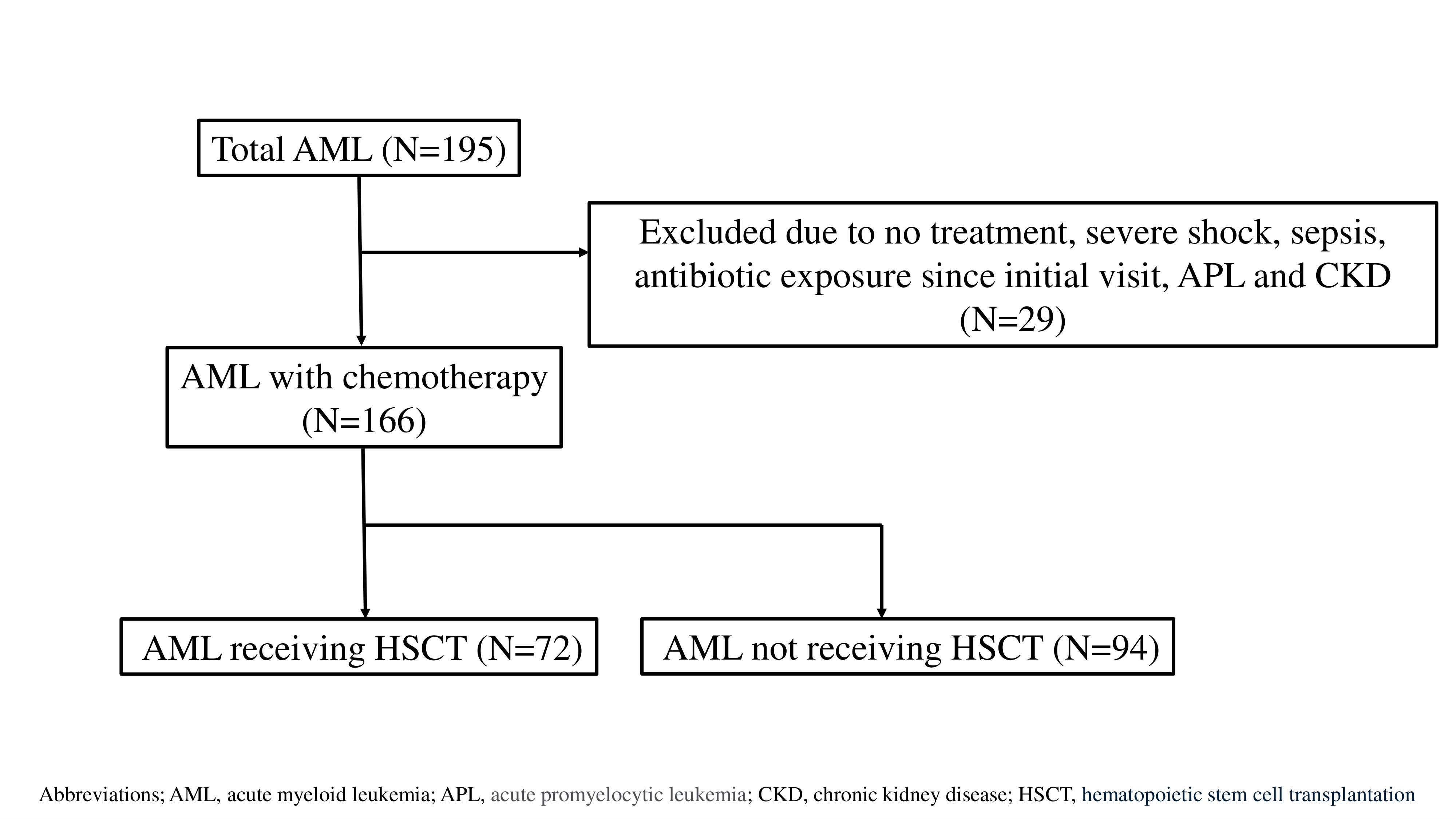

Supplement: Supplementary file 1 — Figure S1: Study flow chart illustrating the patient enrollment process. Figure S2: Receiver operating curve analysis to determine the optimal cutoff for lysozyme to predict AKI (KDIGO > 0). Figure S3: OS (A) of entire patients. OS, overall survival. Figure S4: OS of entire patients according to the positivity of 2017 ELN/2022 ELN adverse risk at first visit. OS, overall survival; ELN, European Leukemia Network. Figure S5: The box plot of the temporal creatinine clearance changes in all patients. Figure S6: OS (A) and PFS (B) of transplant‐ineligible patients according to the first visit serum lysozyme < or ≥ 22.4 μg/mL. OS, overall survival; PFS, progression‐free survival. Figure S7: OS (A), PFS (B), cumulative relapse rate (C), and NRM (D) of transplant‐eligible patients. OS, overall survival; PFS, progression‐free survival; NRM, non‐relapse mortality. Figure S8: The box plot of the temporal serum creatinine changes in transplant‐eligible patients. Table S1: Clinical characteristics of transplant‐eligible patients according to the first visit serum lysozyme ≥ 22.4 μg/mL or not. [file CAM4-15-e71741-s001.zip › cam471741-sup-0001-FigureS1.jpg]

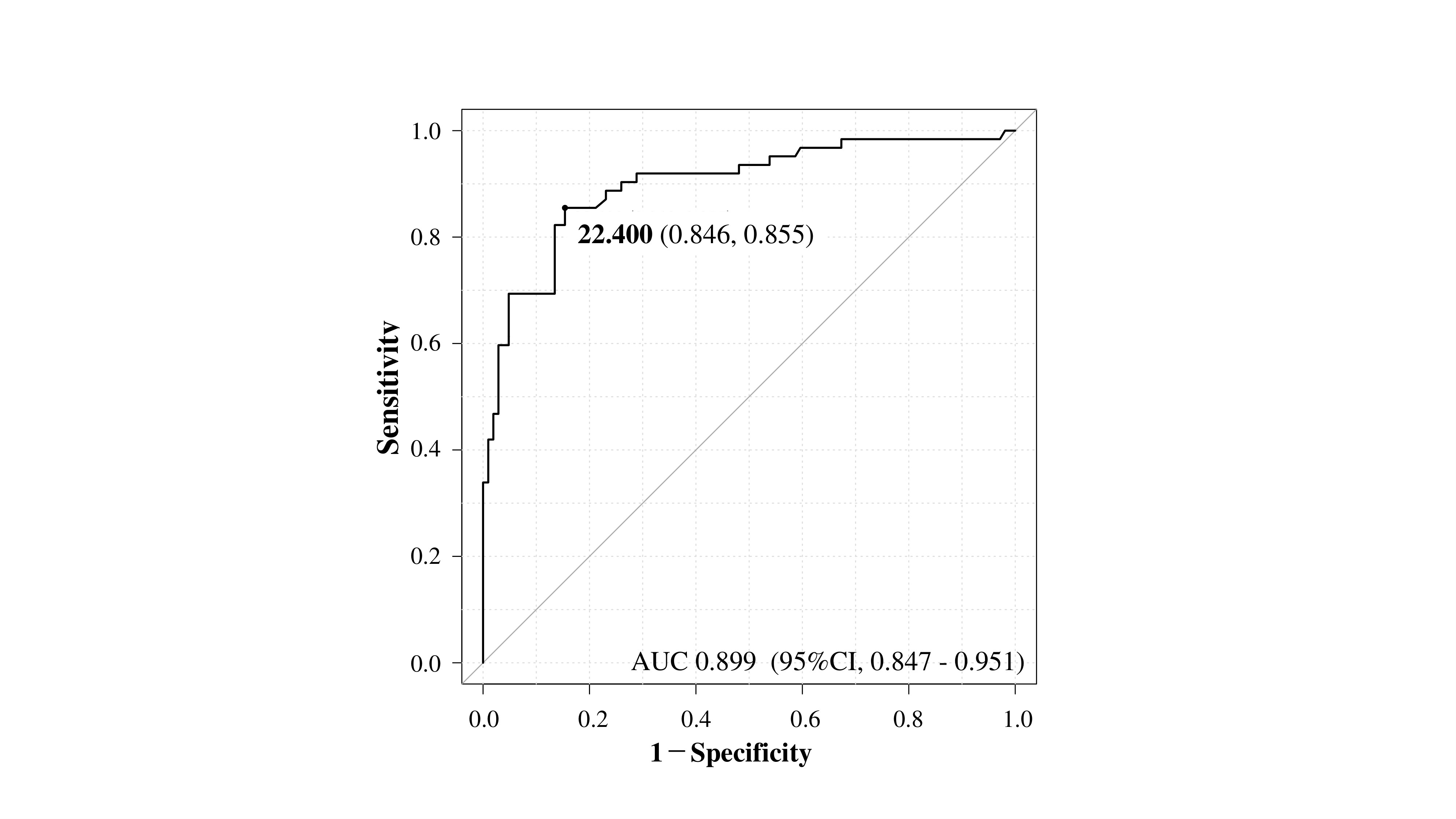

Supplement: Supplementary file 1 — Figure S1: Study flow chart illustrating the patient enrollment process. Figure S2: Receiver operating curve analysis to determine the optimal cutoff for lysozyme to predict AKI (KDIGO > 0). Figure S3: OS (A) of entire patients. OS, overall survival. Figure S4: OS of entire patients according to the positivity of 2017 ELN/2022 ELN adverse risk at first visit. OS, overall survival; ELN, European Leukemia Network. Figure S5: The box plot of the temporal creatinine clearance changes in all patients. Figure S6: OS (A) and PFS (B) of transplant‐ineligible patients according to the first visit serum lysozyme < or ≥ 22.4 μg/mL. OS, overall survival; PFS, progression‐free survival. Figure S7: OS (A), PFS (B), cumulative relapse rate (C), and NRM (D) of transplant‐eligible patients. OS, overall survival; PFS, progression‐free survival; NRM, non‐relapse mortality. Figure S8: The box plot of the temporal serum creatinine changes in transplant‐eligible patients. Table S1: Clinical characteristics of transplant‐eligible patients according to the first visit serum lysozyme ≥ 22.4 μg/mL or not. [file CAM4-15-e71741-s001.zip › cam471741-sup-0002-FigureS2.jpg]
